# The dawdle, dally and delay of diabetic ketoacidosis: Decoding the emergency department length of stay – A chart review

**DOI:** 10.4102/jcmsa.v3i1.200

**Published:** 2025-06-13

**Authors:** Raees Gangat, Nicholas J. Dufourq, Duncan M. Havenga

**Affiliations:** 1 Department of Emergency Medicine, Faculty of Medicine, University of KwaZulu-Natal, Durban, South Africa

**Keywords:** diabetes, diabetic ketoacidosis, length of stay, emergency department, time to resolution

## Abstract

**Background:**

Diabetic ketoacidosis is a life-threatening complication requiring prompt intervention. Understanding factors influencing the duration of emergency department (ED) stay is essential for optimising patient care and resource utilisation.

**Methods:**

A retrospective review was conducted on patients diagnosed with diabetic ketoacidosis at a regional hospital in KwaZulu-Natal between March 2022 and March 2023. Demographics, precipitants, severity, potassium levels and time to resolution were analysed to determine correlations between these variables and the duration of stay in the ED.

**Results:**

Of the 156 records, 105 met the inclusion criteria. The mean age was 36.8 years, with 51.4% male and 98.1% of black ethnicity. Poor compliance (37.1%) and infection (31.4%) were the commonest precipitants. Longer durations in the ED (16.2 h, *p* = 0.007) were linked to cases with unknown precipitants. The median stay was 6 h–12 h, with infection linked to the longest mean duration (10.1 h). Recurrent episodes occurred in 38.1% of patients. Severe diabetic ketoacidosis (13.0 hours, *p* = 0.001) and a lower pH at presentation (*β* = –19.6, *p* < 0.001) were significantly associated with prolonged time to resolution. Potassium abnormalities (29.5%) had no significant effect on duration of stay (*p* = 0.775).

**Conclusion:**

Unknown precipitants significantly influenced the length of stay in the ED. Infection, severe acidosis, and hypokalaemia contributed with variable significance, highlighting that targeted interventions may potentially reduce congestion in these settings.

**Contribution:**

This study provides valuable insights into factors influencing ED length of stay for diabetic ketoacidosis patients, offering evidence to improve clinical management and resource allocation in regional hospitals.

## Introduction

The rising prevalence of diabetes mellitus (DM) has intensified diabetic emergencies as a critical public health concern in South Africa (SA).^1,2^ Among these emergencies, diabetic ketoacidosis (DKA) remains a potentially fatal complication, requiring prompt recognition and management.^2,3^ While international guidelines emphasise efficient emergency department (ED) management to ensure favourable outcomes,^2,4^ the contextual challenges within SA, including resource constraints and limited high-care facilities make effective treatment more complex.^5^

The increasing prevalence of DM poses a noteworthy public health concern in SA, particularly because of complications.^6^ Diabetic ketoacidosis requires swift and efficient management to prevent pernicious outcomes; yet, SA’s healthcare system is confronted with resource constraints, making judicious care laborious.

This study, which examines the factors influencing ED length of stay (LOS) for DKA patients, holds significant social value. By identifying factors such as recurrence, precipitants, severity and time to resolution, these findings can inform strategies to advance patient care, optimise resource utilisation, reduce ED congestion and guide focussed interventions. The research may influence healthcare practices and policies, both regionally and nationally, enhancing the overall effectiveness of DKA management.

Existing studies are predominantly derived from high-income countries,^7,8,9^ creating an empty space and knowledge deficit regarding the unique factors influencing LOS in South African hospitals. This analysis provides insight into the contributing factors to the presentation of DKA as well as those affecting ED LOS in a South African resource-constrained setting.

This study was guided by the conceptual framework of healthcare conveyance in resource-limited environments. It highlights the relationship between patient-related factors (e.g. DKA severity, recurrence and precipitants) as well as operational factors (e.g. timeous interventions). By applying this framework, the study envisioned to classify key determinants of ED LOS and suggest areas for further development in clinical proficiency and patient outcomes. These factors include recurrent presentations, precipitating causes, severity of DKA, initial potassium levels and time to resolution. The findings from this study seek to inform clinical strategies to improve patient outcomes, optimise ED resource utilisation and reduce LOS for DKA patients, thereby enhancing overall healthcare delivery.

Through this lens, this study aims to describe several factors, including patient demographics, factors contributing to the development and presentation of DKA among patients admitted through the ED and evaluates the impact of various factors on ED LOS at a regional hospital in KwaZulu-Natal (KZN).

## Research methods and design

This study employed a descriptive retrospective chart review design to assess the factors influencing ED LOS for patients diagnosed with DKA at a rural hospital ED in KZN, SA. It covered a 12-month period from March 2022 to March 2023.

The study was conducted at a regional hospital located in KZN, SA. The ED at this facility is staffed by emergency physicians and serves a diverse patient population, managing approximately 36 000 patients annually, including trauma, medical and surgical emergencies.

The study involved consecutive sampling, including all patients diagnosed with DKA in the facility during the study period.

### Inclusion criteria

The following are the inclusion criteria for this study:

- Hyperglycaemia – plasma glucose prior to insulin administration > 13.9 mmoL/L
- Acidosis – indicated by blood pH < 7.3 or bicarbonate below 18 mmoL/L
- Ketonaemia – as indicated by blood beta-hydroxybutyrate levels exceeding 3 mmoL/L or urinalysis demonstrating any presence of ketones (because of the limitations of urine dipstick tests in accurately reflecting serum ketone levels)
- Age > 12 years

### Exclusion criteria

The exclusion criteria for this study are as follows:

- Patients diagnosed with hyperglycaemic hyperosmolar state (HHS)
- Age < 12 years
- Simple hyperglycaemia without acidosis
- Patients who refused hospital treatment
- Missing or incomplete records

The determination of the sample size for this study was based on historical data from the ED, showing an average of 13 DKA presentations per month, which equated to an estimated annual sample size of 156 patients. As the aim of the study was to identify risk factors associated with LOS, a sample size of 55 patients was deemed appropriate, considering a medium effect size of 0.15 and 80.0% statistical power.

In the pursuit of a rigorous scientific inquiry, a purpose-designed data collection spreadsheet was meticulously constructed to capture essential variables and methodically record pertinent data extracted from patients’ medical records. The ensuing statistical analysis, integral to unveiling meaningful insights, was executed in collaboration with a statistician.

The variable denoting LOS was subjected to a judicious categorisation scheme, providing nuanced insights into temporal dynamics. Specifically, LOS was stratified into discrete intervals: short stay (0 h – 6 h), medium stay (6 h – 12 h) and long stay (> 12 h). This categorisation ensured a comprehensive understanding of temporal variations in patient disposition. It was selected based on a combination of local ED workflow expectations and resource pressures in acute care settings. While not directly derived from international guidelines, it aligns with locally appropriate time-sensitive care and facilitates meaningful stratification of patient flow and delays.^5^

The phenomenon of multiple presentations was characterised into three distinct categories: no recurrence, single recurrence or multiple recurrences. This classification afforded granularity in assessing the recurrence patterns of patients with DKA. Classification was based on documented medical history available in the patients’ records at this facility. A ‘single recurrence’ referred to one prior documented episode of DKA, while ‘multiple recurrences’ indicated more than one previous episode. No specific timeframe was applied; classification was based solely on the number of past presentations, regardless of the interval between them.

The identification of precipitants, crucial in elucidating causal factors, was categorised into distinct aetiological classes: infection, poor compliance, inadequate dose, alcohol-induced pancreatitis, undiagnosed DM, herbal medication and unknown precipitant. This classification scheme aimed to unravel the multifaceted triggers contributing to DKA.

Infection was defined as a suspected or confirmed infectious trigger for DKA, based on clinical documentation, supported by history, examination and relevant investigations such as radiography, urine dipsticks, ultrasound and elevated inflammatory markers (e.g. white cell count and C-reactive protein). Microbiological testing (microscopy, culture and sensitivity), performed by admitting disciplines, did not reveal initial ED diagnoses.

Poor compliance referred to documented non-adherence to diabetes management, including missed insulin doses, irregular clinic attendance or dietary lapses, based on a patient’s history or provider notes. Inadequate dose described cases where patients were reportedly adherent, but insulin doses were judged clinically insufficient, with provider documentation indicating suboptimal titration or adjustment preceding the DKA episode.

In cases of alcohol-induced pancreatitis, DKA was distinguished from alcoholic ketoacidosis (AKA) based on clinical judgement and documented laboratory data. A diagnosis of DKA was supported by a history of diabetes, significant hyperglycaemia (> 13.9 mmoL/L), and metabolic acidosis with ketonaemia or ketonuria. Alcoholic ketoacidosis was considered less likely under these conditions as it usually presents with normal or low glucose.^10^ In ambiguous cases, the clinician’s recorded diagnosis guided classification.

Severity assessment was conducted using a scoring system derived from key physiological parameters, namely pH, bicarbonate (HCO_3_) and mental state. This scoring system aligned with established guidelines from the Society for Endocrinology, Metabolism and Diabetes of South Africa (SEMDSA), ensuring a standardised approach to severity characterisation.^1^

Furthermore, the initial potassium level on arrival was meticulously stratified into low (< 3.5), normal (3.5–5.5), and high (> 5.5) categories. This stratification enhanced the discernment of potassium dynamics, a critical aspect in DKA management.

Lastly, time to resolution was governed by resolution of diagnostic criteria for DKA, which included pH > 7.3, serum glucose < 13.9 mmoL/L, absent ketonuria and bicarbonate > 18 mmoL/L. Blood gas parameters including pH and bicarbonate were measured 2–4 hourly, whereas glucose and ketonuria were measured 1–2 hourly.

In summation, this systematic categorisation strategy, guided by empirical and clinical considerations, forms the backbone of the statistical analysis, ensuring a robust and nuanced exploration of the collected data.

A data collection spreadsheet was used to record the data, with double-entry verification by the principal investigator to ensure accuracy. Data were stored in a password-protected Microsoft Excel document.^11^

Data analysis was performed using descriptive statistics to summarise both categorical and continuous variables. Categorical variables were described using frequencies and percentages, while continuous variables were summarised as mean ± standard deviation (s.d.) or median and interquartile range, depending on their distribution. Time-related variables, such as LOS and time to resolution, were expressed in hours.

The analysis focussed on several key aspects, including LOS categorisation (short stay: 0 h – 6 h, medium stay: 6 h – 12 h and long stay: > 12 h), recurrent presentations (no recurrence, single recurrence and multiple recurrences) and precipitating factors (infection, poor compliance, inadequate dosing, alcohol-induced pancreatitis, undiagnosed DM, herbal medication and unknown precipitants).

A standardised DKA protocol was followed in the ED in KZN. Initial fluid resuscitation included a 1 L bolus of balanced crystalloid solution (modified Ringer’s lactate or plasmalyte B), followed by a maintenance rate at 250 mL/h – 500 mL/h, adjusted based on haemodynamic status and urine output. Short-acting insulin was infused at 0.1 units/kg/h, titrated to achieve a glucose reduction of 3 mmoL/L/h and HCO_3_ rise > 1 mmoL/L/h. Potassium replacement was guided by serum levels. If K^+^ was less than 3.5 mmoL/L, 40 mmol potassium phosphate (KPO_4_) was added to the balanced crystalloid infusion and insulin was withheld. If K^+^ was between 3.5 and 5.5 mmoL/L, 20 mmoL of KPO_4_ was added to the balanced crystalloid, and if K^+^ was greater than 5.5 mmoL/L, replacement was withheld. Glucose was monitored hourly and venous blood gases (including electrolytes) every 2 h – 4 h. If glucose fell below 15 mmoL/L, the maintenance fluid rate was halved and 10% dextrose water initiated at 125 mL/h (if glucose < 15 mmoL/L) and 250 ml/h (if glucose < 10 mmoL/L). Protocol adherence was consistent, with minor deviations noted in cases of severe hypokalaemia.

All statistical analyses were conducted using IBM SPSS Statistics version 30.^12^

### Ethical considerations

Ethics approval was granted from the Biomedical Research Ethics Committee, University of KZN (No. BREC/00007238/2024) and the Institutional Review Board of a rural hospital in KZN, as well as from the Department of Health KZN (NHRD REF: KZ_202407_007). All patient data were de-identified to ensure confidentiality and privacy. Patient participation and informed consent was not required because of the retrospective nature of the study. All data were handled in strict accordance with ethical guidelines to maintain patient confidentiality.

## Results

**FIGURE 1:**
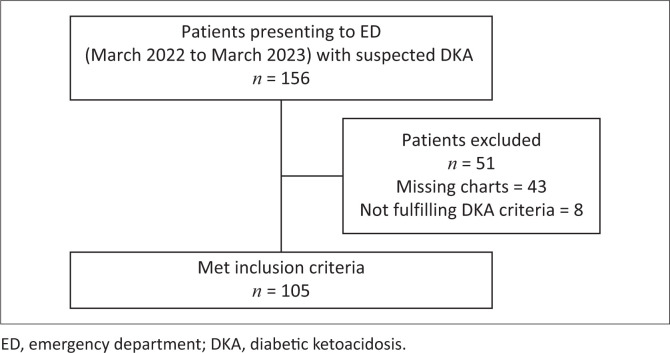
Study sampling breakdown.

A total of 156 patients were identified from triage registers initially, with a total of 105 patients included in the analysis as depicted in Figure 1. The mean age was 36.8 years (s.d. = 17.9). The age, sex, ethnic group and diabetes type distribution are depicted in Table 1. Referral sources were diverse, with self-presentations comprising 41 cases (39.0%) and the remaining 64 cases (61.0%) were distributed across 22 referral facilities.

**TABLE 1:** Demographic data.

Demographics	Variables	Counts	%
Age (years)	12–20	24	22.9
	20–30	20	19.1
	30–40	18	17.1
	50–60	17	16.2
	40–50	13	12.4
	60–70	10	9.6
	> 70	3	2.9
Sex	Male	54	51.4
	Female	51	48.6
Race	Black people	103	98.1
	White people	2	1.9
DM	Type 1	52	49.5
	Type 2	53	50.5

DM, diabetes mellitus.

**FIGURE 2:**
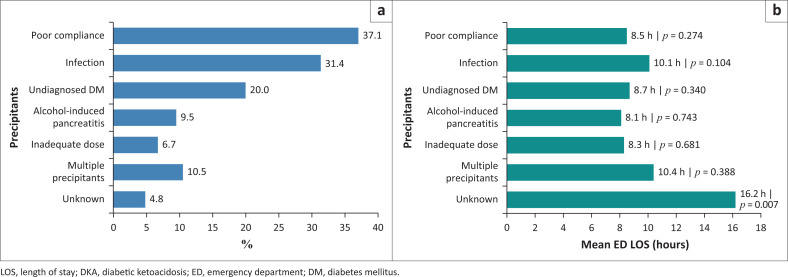
Prevalence and mean length of stay of diabetic ketoacidosis precipitants: (a) Prevalence of DKA precipitants, (b) Mean ED LOS by precipitants.

The frequency of presentations per precipitant of DKA is depicted in Figure 2. The total percentage exceeds 100.0% because certain cases had multiple precipitants recorded. For example, a single patient could present with both ‘infection’ and ‘poor compliance’ as precipitating factors. Therefore, the percentages represent the frequency of each precipitant as a proportion of the total sample, but some cases are tallied more than once because of multiple contributing factors. This was evident in 11 cases in this cohort.

The analysis explored the association between different DKA precipitants and ED LOS. The results are summarised in Figure 2. The *β*-coefficient (in the known precipitant group) for infection was the largest at +2.0, indicating that infection is the most significant predictor of increased ED LOS compared to the reference group (alcohol-induced pancreatitis). Smaller positive coefficients were observed for other precipitants including ‘undiagnosed DM’ (+0.6), ‘poor compliance’ (+0.4) and ‘inadequate dose’ (+0.2). Patients with an unknown precipitant had a longer LOS in comparison.

Recurrent DKA presentations were categorised as no recurrence, a single recurrence or multiple recurrences, with prevalences of 61.9% (mean ED LOS: 8.6 h, s.d.: 4.7), 15.2% (mean ED LOS: 10 h, s.d.: 4.3) and 22.9% (mean ED LOS: 10.1 h, s.d.: 4.3), respectively. Overall, 38.1% of the 105 patients experienced at least one recurrent episode during the study period. There were no statistically significant differences in ED LOS across recurrence groups (*p* = 0.065).

Diabetic ketoacidosis severity was classified according to the SEMDSA severity classification score.^1^ Severe, moderate and mild DKA accounted for 37.1% (mean ED LOS: 9.4 h, s.d.: 4.6), 43.8% (mean ED LOS: 9 h, s.d.: 4.0) and 19.1% (mean ED LOS: 9.1 h, s.d.: 5.6) of cases, respectively. There were no significant differences in ED LOS across the severity groups (*p* = 0.742).

Initial serum potassium levels were classified as low (≤ 3.5 mmoL/L), normal (3.5 mmoL/L – 5.5 mmol/L) or high (> 5.5 mmoL/L). Hypokalaemia was observed in 6.7% of patients (mean ED LOS: 12.3 h, s.d.: 4.7), normal potassium in 70.5% (mean ED LOS: 8.6 h, s.d.: 3.6) and hyperkalaemia in 22.9% (mean ED LOS: 9.4 h, s.d.: 4.1). There was no statistically significant difference in ED LOS between groups (*p* = 0.775).

Table 2 depicts the categorical comparisons between ED LOS and various factors where LOS was stratified into three discrete intervals to provide insights into temporal dynamics. These three intervals were categorised into short stay (25.7%), medium stay (51.4%) and long stay (23.0%) of the total sample size of 105 patients, respectively.

**TABLE 2:** Categorical comparisons of emergency department length of stay.

Variable	ED length of stay (hours)	*n*	Age (years)	Time to resolution (hours)	pH	HCO_3_^+^ (mmol/L)	K^+^ (mmol/L)	Glucose (mmol/L)	PCO_2_ (mmHg)
Mean	0–6	27	33.900	9.900	7.200	11.600	4.700	23.000	22.400
	6–12	54	38.200	10.600	7.100	11.100	5.000	25.300	26.400
	> 12	24	36.800	12.300	7.100	11.200	4.900	23.900	24.500
*p*-value	-	-	0.591	0.264	0.318	0.940	0.775	0.846	0.330

ED, emergency department.

There was no statistically significant correlation between time to resolution and ED LOS (*r* = 0.12, *p* = 0.210), indicating that changes in time to resolution were not associated with corresponding increases or decreases in ED LOS.

Although not part of the original study aims, time to resolution was additionally analysed as a secondary outcome to explore potential associations with DKA severity and metabolic parameters. Complete resolution of metabolic parameters occurred in 51.4% of cases within the ED, with a mean time to resolution of 10.8 h (s.d.: 6.9). Among patients who did not achieve resolution in the ED, 48.6% required admission for further management. Time to resolution varied significantly by severity, with mild cases resolving faster (mean = 6.1 h, s.d. = 5.0) than moderate (mean = 11.0 h, s.d. = 6.3) and severe cases (mean = 13.0 h, s.d. = 7.2), *p* = 0.001. Furthermore, cases that achieved complete resolution within the ED had a significantly shorter resolution time (mean = 8.8 h, s.d. = 5.8) compared to cases with only partial resolution prior to disposition (mean = 12.6 h, s.d. = 7.2), *p* = 0.004.

A significant correlation was observed between time to resolution and metabolic parameters including pH (*r* = −0.44, *p* < 0.001), HCO3 (*r* = −0.50, *p* < 0.001) and PCO2 (*r* = −0.30, *p* = 0.002), which were all negatively correlated with time to resolution, indicating that lower values of these parameters were associated with prolonged metabolic recovery.

In linear regression analysis, pH at presentation was identified as a significant predictor of prolonged time to resolution, with a coefficient of *β* = −19.6 (95% CI: −27.8 to −11.4), *p* < 0.001. This suggests that for every 0.1 unit increase in pH, time to resolution decreased by approximately 19.6 h. However, HCO_3_ (*β* = −0.8, *p* < 0.001) and PCO_2_ (*β* = −0.2, *p* = 0.005), had less of an impact but were still statistically significant.

A regression analysis was also conducted to observe the impact of disease severity on time to resolution, using mild severity as the reference category. The results indicated that in comparison to mild cases, moderate severity was associated with a 4.9 h increase in time to resolution (*p* = 0.005). Moreover, severe cases demonstrated an even greater delay, with a 7 h increase in time to resolution (*p* < 0.001). However, the multiple regression analysis only showed a significance with pH (*p* = 0.038).

## Discussion

The demographic trends in this study align with global trends of DKA prevalence among younger adults, with a mean age of 36.8 years and nearly analogous sex distribution (51.4% male, 48.6% female).^13^

Global patterns revealed type 1 diabetic patients having a greater likelihood of developing DKA because of complete insulin deficiency, but this study suggests a relatively even distribution of prevalence between both types.^14^ In contrast, type 2 diabetes cases with DKA are often linked to severe precipitating factors such as infections or stress events.^15^ The high proportion of referrals encompassing 61.0% of patients in this cohort may indicate a deficit in recognition or transfer from a primary care setting, a critical flaw in the care continuum.

This study outlines infection as a primary contributor to prolonged ED LOS. The mean stay was 10.1 h with a positive regression coefficient (*β* = +2.0); however, this was not statistically significant (*p*-value = 0.104). Findings have been reported in preceding studies, where infections exacerbate metabolic derangements, delay resolution and complicate treatment pathways.^3,16^ Addressing infections early through enhanced screening and targeted therapies remains critical to reducing hospital resource utilisation.

While poor compliance (37.1%) and undiagnosed DM (20.0%) were also noteworthy contributors, their association with ED LOS was comparatively lower (8.5 h and 8.7 h, respectively). Studies have consistently shown poor compliance as an avoidable precipitant of DKA, linked to a rift in diabetes education, follow-up care and socioeconomic barriers.^17,18^ Prominently, unknown precipitants were associated with the longest ED LOS (16.2 h, *p* = 0.007), indicating diagnostic doubt and delays in proceeding with appropriate treatment, an issue in restricted resource settings.

The remarkable difference observed between patients with known and unknown precipitants further stresses the importance of precipitant identification. Patients with unknown precipitants had a suggestively longer ED LOS (mean = 16.2 h), as revealed by the regression analysis (*β* = 7.4, *p* < 0.001). The delay likely stems from the need for supplementary investigations and the difficulties in initiating treatment without a clear precipitating factor. From a clinical perspective, these findings suggest a twofold necessity: Firstly, to consider preventing modifiable precipitants such as poor compliance and inadequate dosing through education and follow-up care, and secondly to improve early diagnostics and management of infections to reduce ED stays. Furthermore, approaches to streamline diagnostic roadmaps and identifying unknown precipitants swiftly could have a meaningful impact on reducing delays in care.

Overall, this study outlines the diverse contributions of DKA precipitants to ED LOS, with infection being the most significant. Addressing these factors could augment resource utilisation and improve patient outcomes in the ED.

Recurrent DKA presentations were observed in 38.0% of the cohort, highlighting a substantial burden on both patients and emergency care services. While single recurrences may reflect isolated lapses in diabetes management, multiple recurrences often suggest deeper, systemic issues requiring sustained, multidisciplinary intervention. Even though patients with recurrent presentations had a longer mean ED LOS (11.4 h) compared to those without recurrence (8.3 h), this difference was not statistically significant (*p* = 0.605). Nonetheless, the increased resource utilisation underscores the importance of identifying and addressing modifiable risk factors – such as poor treatment adherence and inadequate outpatient follow-up – to reduce recurrence rates and improve long-term outcomes.

Diabetic ketoacidosis severity did not significantly influence ED LOS (*p* = 0.742); however, severe cases were associated with extended time to resolution (13 h) compared to mild cases (6.1 h, *p* = 0.001). This is consistent with local studies showing that disease worsening severity correlates with delayed metabolic recovery.^19^ Notably, lower pH at presentation emerged as a significant predictor of prolonged resolution time (*β* = −19.6, *p* < 0.001), highlighting the relationship between severe acidosis and recovery delays.^20,21^ Early triage and prompt management of severe cases are crucial to improving patient outcomes and reducing ED burden.

While hypokalaemia (K+ ≤ 3.5 mmoL/L) was associated with the longest mean ED LOS (12.3 h, s.d.: 4.7) and hyperkalaemia (K+ > 5.5 mmoL/L) had a mean ED LOS of 9.4 h (s.d.: 4.1), normal potassium levels (K+ 3.5 mmoL/L – 5.5 mmoL/L) were associated with the shortest mean ED LOS of 8.6 h (s.d.: 3.6). While no statistically significant differences were observed between the potassium level groups (*p* = 0.775), the trend towards longer resolution times in hypokalaemia suggests that cautious potassium replacement may contribute to the extended ED stay. This highlights the importance of early correction of electrolyte disturbances to avoid potential complications.

Previous studies have similarly reported trends where hypokalaemia complicates DKA management because of the need for cautious potassium replacement to prevent cardiac arrhythmias.^22^

Although potassium levels showed some variation across ED LOS categories, other biochemical factors, including pH, HCO_3_, glucose and PCO_2_, did not demonstrate statistically significant differences in their relationship to ED LOS (Table 2). Similarly, neither patient age nor time to resolution significantly influenced ED LOS. These findings suggest that while metabolic correction is a key component of DKA management, other clinical and operational factors will likely play an influential role in determining ED LOS.^23^ The absence of a clear correlation between these variables and ED LOS highlights the complexity of patient flow and decision-making in the ED,^24^ reinforcing the need for a multifactorial approach when evaluating delays in patient disposition.

These findings establish the interdependent relationship between these variables, emphasising the need for all-inclusive and individualised management strategies. Addressing these factors through early intervention, rapid electrolyte correction, and efficient care pathways could markedly reduce ED LOS and improve outcomes for DKA patients. The stratified analysis of ED LOS provides critical insights into healthcare resource management and patient outcomes, particularly in low-resource settings.

Short-stay patients (≤ 6 h), comprising 25.7% of the cohort, reflect cases where early stabilisation and management were achieved effectively, driven by established streamlined protocols. Medium-stay patients (6 h – 12 h) represented the largest group (51.4%) whose management relied on timeous interventions and resource availability.

In resource-constrained settings, where intensive care unit (ICU) beds and high-care facilities are often scarce,^5^ prolonged LOS in the ED because of delayed transfers can undesirably impact the overall throughput of the ED.^5^ Furthermore, the association of long stays with severe cases highlights the necessity of early identification and triage of high-risk patients to improve outcomes. Cases that achieved complete resolution within the ED had a significantly shorter resolution time in comparison to those who only partially resolved (mean = 8.8 h vs. 12.6 h, *p* = 0.004). This did not translate into a reduced ED LOS and suggests that factors beyond time to resolution influenced overall LOS. These include potential factors mentioned earlier such as disease severity, bed availability and hospital-level operational constraints. It is possible that patients achieving full resolution in the ED represented less severe cases, allowing for faster biochemical correction without necessarily expediting discharge or transfer. Further investigation into these determinants could help clarify their impact on patient flow and resource allocation. Developing focussed strategies to reduce LOS, such as rapid infection management, optimisation of bed allocation and better follow-up care for recurrent DKA patients could significantly enhance both patient outcomes and system efficiency.

The regression analysis findings correspond closely with existing literature, emphasising the significance of initial pH levels in predicting time to resolution for DKA patients. Studies identified lower pH levels as a strong determinant of prolonged metabolic recovery,^25^ revealing the direct relationship between acidosis severity and treatment complexity. The observed regression coefficient (*β* = −19.6, *p* < 0.001) highlights this relationship, showing that each 0.1 unit increase in pH significantly reduces resolution time.

Clinically, these findings advocate for the prioritisation of rapid triage and stabilisation protocols focussed on correcting severe acidosis and addressing underlying infections. Implementing standardised care pathways for DKA, discovered by predictive metrics such as pH levels, could significantly enhance efficiency and patient recovery in resource-constrained settings.

### Methodological challenges and study limitations

The retrospective chart review methodology introduces several inherent challenges and limitations that necessitate consideration. Retrospective chart reviews are susceptible to the absence of crucial data, including lost patient records, poor documentation and illegible handwriting. Parameters such as documented bed availability, poor compliance to medication and incorrect medication use may not be clearly documented because of limitations in history taking. Blood gas results, pivotal for assessing the metabolic status of DKA patients, may be absent from the patient files. The physical loss of hard copy notes from patient files may contribute to data gaps.

Lactate could not be collected routinely as certain blood gas analysers did not include a lactate result. As a result, lactate was inconsistently measured and therefore could not be included in the analysis of factors influencing ED LOS or time to resolution. This limits the ability to assess the potential role of lactic acidosis in this cohort.

Other limitations included diabetes type classification, which was based on information from patients’ medical records. In the absence of routine autoantibody testing to confirm type 1 diabetes, the classification depended on clinical judgement, history of insulin dependency, age at diagnosis and provider documentation.

To address these challenges, a transparent reporting approach was adopted, providing detailed information on the extent and nature of missing data, including quantification, reasoning and detailing the steps taken to address data collection gaps. During the capturing process, poor documentation and filing were apparent, which involved using multiple sources including inpatient ICU and high-care records, outpatient files, triage and discharge registers, ICU electronic data base and nursing transfer entries to ensure all parameters were captured. Cross referencing discharge entries and nursing transfers to ensure accurate timings were recorded was also conducted. The transparency in reporting will enable readers to assess the potential impact of missing data on the study’s results and conclusions. In addition, sensitivity analysis was conducted to evaluate the robustness of the data and identify any biases resulting from missing information.

### Recommendations

The implications of these findings extend to public health and policy, particularly in resource-constrained settings. Attending to the high prevalence of infection-related DKA and recurrent admissions necessitates systemic improvements in outpatient diabetes care, including enhanced education and accessibility to medications. Furthermore, stratagems to diminish ED congestion through optimised resource allocation, such as rapid triage systems and enhanced ICU accessibility, could meaningfully improve healthcare delivery. This can be adopted in the early diagnostic phase as we have noticed that unknown precipitants had a statistically significant effect on ED LOS, and pH levels had a statistically significant effect on time to resolution. Using these parameters to trigger an early ICU consultation can lead the way in quality improvement and produce a significant difference in ED resource management of DKA. These interventions not only improve patient outcomes but also assuage the burden on overstrained emergency services, paving the way for more sustainable healthcare systems in under-resourced regions.

The findings highlight the need for focussed interventions to address infection-related DKA, improve compliance to diabetes care and optimise management of severe cases. Diabetic ketoacidosis patients often require high care or ICU beds, leading to prolonged stays in the ED. This contributes to challenges such as overcrowding, often driven by access block. Despite the availability of specialised departments such as internal medicine and critical care, DKA patients may remain in the ED for extended periods awaiting disposition to appropriate wards. Systemic strategies, including enhanced outpatient follow-up, diabetes education programmes and standardised care protocols are crucial to reducing DKA recurrence and alleviating ED congestion. Early identification and aggressive management of severe acidosis, electrolyte imbalances and infection can significantly improve recovery times and resource utilisation.

## Conclusion

This study provides a broad analysis of the demographic features, number of presentations, precipitating factors and clinical parameters influencing ED LOS in patients with DKA. Stratifying LOS into short, medium and long categories provided nuanced insights, with factors such as infection, severe acidosis and hypokalaemia contributing to prolonged ED stays with variable significance. Patients with unknown precipitants contributed significantly to prolonged ED stays. The regression analysis highlighted the significant role of pH as a predictor of time to resolution, emphasising the need for early stabilisation of acidosis to optimise outcomes. These findings emphasise the need for additional research in the future accompanied by targeted quality improvement strategies to improve DKA management and reduce ED congestion.

## References

[1] Amod A. Society for Endocrinology, Metabolism and Diabetes of South Africa (SEMDSA) 2017 guidelines for the management of type 2 diabetes mellitus. J Endocrinol Metabol Diabetes South Afr. 2017;22(1):S64.

[2] Umpierrez GE, Davis GM, ElSayed NA, et al. Hyperglycemic crises in adults with diabetes: A consensus report. Diabetes Care. 2024;47(8):1257–1275. 10.2337/dci24-0032 39052901 PMC11272983

[3] Ndebele NFM, Naidoo M. The management of diabetic ketoacidosis at a rural regional hospital in KwaZulu-Natal. Afr J Prim Health Care Fam Med. 2018;10(1):e1–e6. 10.4102/phcfm.v10i1.1612 PMC5913763 29781681

[4] Carter EJ, Pouch SM, Larson EL. The relationship between emergency department crowding and patient outcomes: A systematic review. J Nurs Scholarsh. 2014;46(2):106–115. 10.1111/jnu.12055 24354886 PMC4033834

[5] Mashao K, Heyns T, White Z. Areas of delay related to prolonged length of stay in an emergency department of an academic hospital in South Africa. Afr J Emerg Med. 2021;11(2):237–241. 10.1016/j.afjem.2021.02.002 33747758 PMC7966966

[6] Gangakhedkar G. Diabetic ketoacidosis and intensive care. J Res Innov Anesth. 2019;4:29–31. 10.5005/jp-journals-10049-0072

[7] LM L. Games people play: Lessons on performance measure gaming from New Zealand comment on ‘Gaming New Zealand’s emergency department target: How and why did it vary over time and between organisations?. Int J Health Policy Manag. 2021;10(4):225–227. 32610791 10.34172/ijhpm.2020.41 PMC8167274

[8] Geelhoed GC, De Klerk NH. Emergency department overcrowding, mortality and the 4-hour rule in Western Australia. Med J Aust. 2012;196(2):122–126. 10.5694/mja11.11159 22304606

[9] Weber E, Mason S, Freeman J, Coster J. Implications of England’s four-hour target for quality of care and resource use in the emergency department. Ann Emerg Med. 2012;60(6):699–706. 10.1016/j.annemergmed.2012.08.009 23102917

[10] Brutsaert EF. Alcoholic ketoacidosis MSD manual professional edition: MSD manual. New Jersey: Merck & Co.; 2023.

[11] Microsoft. Microsoft Excel. 2307 ed. Washington, USA: Microsoft Corporation; 2016.

[12] IBM. IBM SPSS statistics. 30 ed. Armonk: IBM Corp; 2023.

[13] Barski L, Harman-Boehm I, Nevzorov R, et al. Gender-related differences in clinical characteristics and outcomes in patients with diabetic ketoacidosis. Gend Med. 2011;8(6):372–377. 10.1016/j.genm.2011.09.032 22055610

[14] Wang ZH, Kihl-Selstam E, Eriksson JW. Ketoacidosis occurs in both Type 1 and Type 2 diabetes – A population-based study from Northern Sweden. Diabet Med. 2008;25(7):867–870. 10.1111/j.1464-5491.2008.02461.x 18644074

[15] Lotter N, Lahri S, Van Hoving DJ. The burden of diabetic emergencies on the resuscitation area of a district-level public hospital in Cape Town. Afr J Emerg Med. 2021;11(4):416–421. 10.1016/j.afjem.2021.05.004 34703733 PMC8524109

[16] Barski L, Nevzorov R, Rabaev E, et al. Diabetic ketoacidosis: Clinical characteristics, precipitating factors and outcomes of care. Isr Med Assoc J. 2012;14(5):299–303. 22799061

[17] Randall L, Begovic J, Hudson M, et al. Recurrent diabetic ketoacidosis in inner-city minority patients: Behavioral, socioeconomic, and psychosocial factors. Diabetes Care. 2011;34(9):1891–1896. 10.2337/dc11-0701 21775761 PMC3161256

[18] Nam S, Chesla C, Stotts NA, Kroon L, Janson SL. Barriers to diabetes management: Patient and provider factors. Diabetes Res Clin Pract. 2011;93(1):1–9. 10.1016/j.diabres.2011.02.002 21382643

[19] Thomas S, Mohamed NA, Bhana S. Audit of diabetic ketoacidosis management at a tertiary hospital in Johannesburg, South Africa. S Afr Med J. 2019;109(6):407–411. 10.7196/SAMJ.2019.v109i6.13700 31266558

[20] AlWahbi MF, Alharbi SH, Almesned SA, et al. An audit of factors impacting the time to resolution of the metabolic parameters in diabetic ketoacidosis patients. Cureus. 2022;14(11):e31142. 10.7759/cureus.31142 36505109 PMC9728988

[21] Lee MH, Calder GL, Santamaria JD, Maclsaac RJ. Diabetic ketoacidosis in adult patients: An audit of factors influencing time to normalisation of metabolic parameters. Intern Med J. 2018;48(5):529–534. 10.1111/imj.13735 29316133

[22] Arora S, Cheng D, Wyler B, Menchine M. Prevalence of hypokalemia in ED patients with diabetic ketoacidosis. Am J Emerg Med. 2012;30(3):481–484. 10.1016/j.ajem.2011.01.002 21316179

[23] Rose L, Scales DC, Atzema C, et al. Emergency department length of stay for critical care admissions. A population-based study. Ann Am Thorac Soc. 2016;13(8):1324–1332. 10.1513/AnnalsATS.201511-773OC 27111127

[24] Arkun A, Briggs WM, Patel S, Datillo PA, Bove J, Birkhahn RH. Emergency department crowding: Factors influencing flow. West J Emerg Med. 2010;11(1):10–15. 20411067 PMC2850834

[25] Sabry AA, Alkafafy AM, Morsy EY, Aiad A, Montasser M. Factors affecting time to recovery from diabetic ketoacidosis in adult diabetic patients in Alexandria Main University Hospital. Egypt J Intern Med. 2024;36(1):99. 10.1186/s43162-024-00365-x

